# Effect of Ethiopia’s Health Extension Program on Maternal and Newborn Health Care Practices in 101 Rural Districts: A Dose-Response Study

**DOI:** 10.1371/journal.pone.0065160

**Published:** 2013-06-04

**Authors:** Ali Mehryar Karim, Kesetebirhane Admassu, Joanna Schellenberg, Hibret Alemu, Nebiyu Getachew, Agazi Ameha, Luche Tadesse, Wuleta Betemariam

**Affiliations:** 1 The Last Ten Kilometers Project, JSI Research & Training Institute, Inc., Addis Ababa, Ethiopia; 2 Federal Democratic Republic of Ethiopia, Ministry of Health, Addis Ababa, Ethiopia; 3 London School of Hygiene & Tropical Medicine, London, United Kingdom; Tulane University School of Public Health and Tropical Medicine, United States of America

## Abstract

**Background:**

Improving newborn survival is essential if Ethiopia is to achieve Millennium Development Goal 4. The national Health Extension Program (HEP) includes community-based newborn survival interventions. We report the effect of these interventions on changes in maternal and newborn health care practices between 2008 and 2010 in 101 districts, comprising 11.6 million people, or 16% of Ethiopia’s population.

**Methods and Findings:**

Using data from cross-sectional surveys in December 2008 and December 2010 from a representative sample of 117 communities (*kebeles*), we estimated the prevalence of maternal and newborn care practices, and a *program intensity score* in each community. Women with children aged 0 to 11 months reported care practices for their most recent pregnancy and childbirth. The *program intensity score* ranged between zero and ten and was derived from four outreach activities of the HEP front-line health workers. Dose-response relationships between changes in program intensity and the changes in maternal and newborn health were investigated using regression methods, controlling for secular trend, respondents’ background characteristics, and community-level factors.

Between 2008 and 2010, median *program intensity score* increased 2.4-fold. For every unit increase in the score, the odds of receiving antenatal care increased by 1.13 times (95% CI 1.03–1.23); the odds of birth preparedness increased by 1.31 times (1.19–1.44); the odds of receiving postnatal care increased by 1.60 times (1.34–1.91); and the odds of initiating breastfeeding immediately after birth increased by 1.10 times (1.02–1.20). *Program intensity score* was not associated with skilled deliveries, nor with some of the other newborn health care indicators.

**Conclusions:**

The results of our analysis suggest that Ethiopia’s HEP platform has improved maternal and newborn health care practices at scale. However, implementation research will be required to address the maternal and newborn care practices that were not influenced by the HEP outreach activities.

## Introduction

Ethiopia is committed to reducing the under-five mortality rate to 68 deaths per 1,000 live births by 2015 in order to achieve Millennium Development Goal four [1]. Between 2000 and 2011, under-five mortality in the country declined dramatically, from 166 to 88 deaths per 1,000 live births [2]. Nevertheless, as in other developing countries, the reduction is mainly a result of fewer deaths in children one to 59 months old, while neonatal (first 28 days of life) mortality has shown more modest change [3], dropping from 49 to 39 deaths per 1,000 live births between 2000 and 2005, and reaching 37 deaths per 1,000 live births in 2011 [2]. Neonatal deaths now account for 63% of all infant deaths and 42% of all under-five deaths. Reducing neonatal mortality is now critical to achieving the 4^th^ Millennium Development Goal [3], [4].

Simple community-based strategies to improve antenatal, childbirth, and newborn health care practices have been shown to reduce neonatal deaths [5]. These community-based strategies include clean delivery practices (clean hands and delivery surface), clean umbilical cord care (cutting the umbilical cord with a sterile instrument, tying it with a sterile thread, and applying nothing to the cut stump of the cord), thermal care (immediate drying and wrapping of the baby after delivery, delay bathing the baby for more than six hours, and skin-to-skin contact with the mother), extra care for low birth weight or preterm birth (additional warmth, cleanliness and nutrition and early recognition of disease), and early and exclusive breastfeeding to minimize the risk factors associated with neonatal mortality in developing countries. Such strategies are ideal for Ethiopia because 90% of births still take place at home [2] and the Health Extension Program (HEP) provides a platform for delivering such strategies.

The HEP was launched in 2003 and aims to provide universal access to primary health care services [6]–[8], mainly preventive, through more than 34,000 government-salaried female health extension workers (HEWs). Two HEWs were placed in a health post to serve a *kebele*, the smallest administrative unit, with about 5,000 people. HEWs spent 75% of their time on outreach activities: conducting household visits, educating families to adopt healthy life-style and serve as ‘model families’ in their neighborhood; and, organizing communities to participate in the expansion of HEP services. A network of volunteers, drawn from ‘model family’ households, supported the HEWs by providing essential health messages to the community [6].

**Figure 1:**
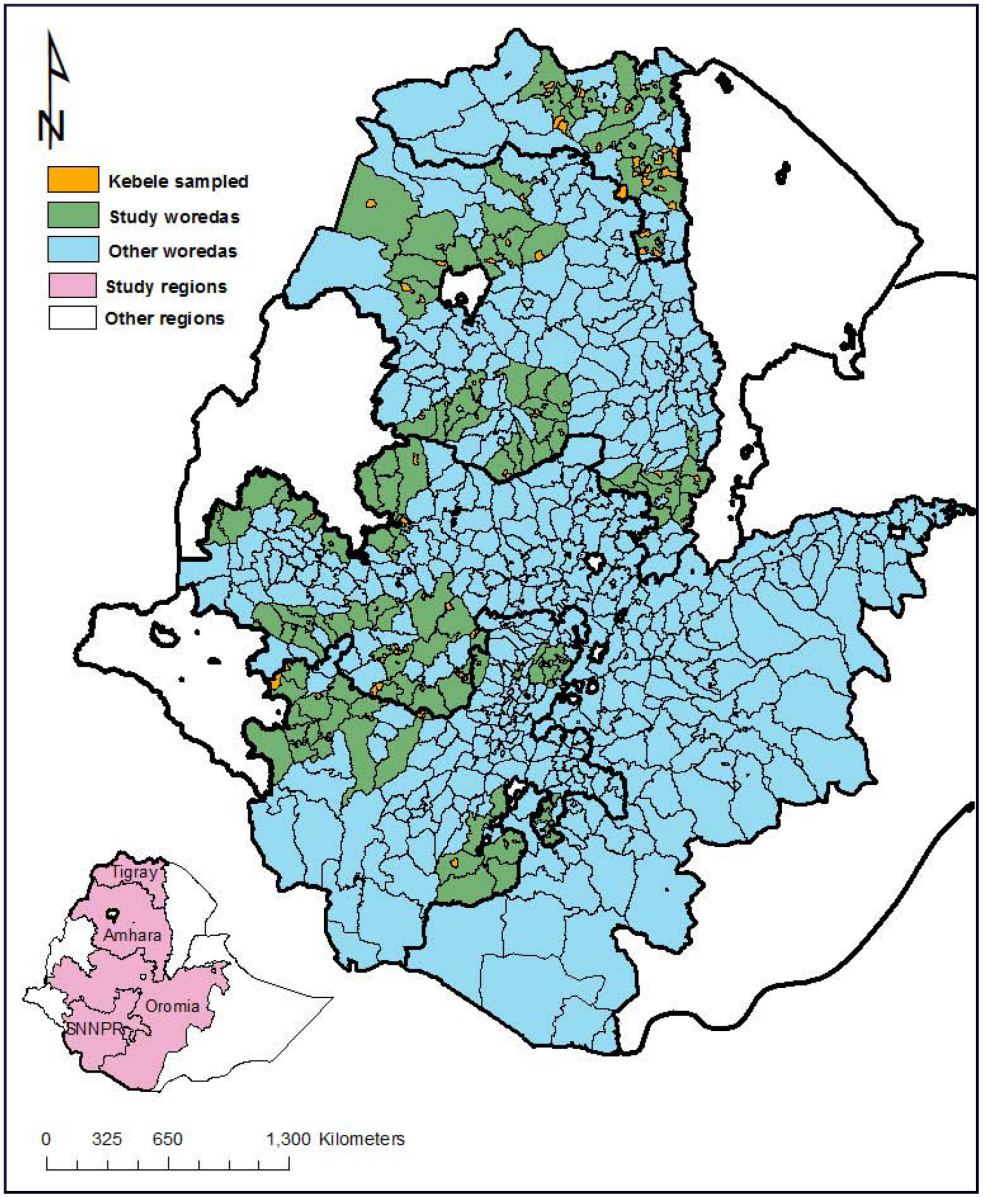
Map of Ethiopia showing the study areas and location of primary sampling units (i.e., kebeles).

Child survival strategies implemented under the HEP included immunization, vitamin A distribution, oral rehydration therapy, distribution of bed nets, anti-malarial, deworming, and child health and nutrition education. Evidence-based essential newborn care including promotion of clean childbirth practices, clean umbilical cord care, thermal care, extra care for low birth weight babies, and early and exclusive breastfeeding [5], [9]–[14], were part of the HEP strategy, yet prior to 2009 the HEWs were not skilled to provide it. A program to equip the HEWs with skills to promote essential newborn care practices was introduced from early 2009 in 101 districts (*woredas*) (Figure 1), through support from the Last Ten Kilometers (L10K) project. This area included about 11.6 million people, approximately 16% of Ethiopia’s population.

To the best of our knowledge, previous published evaluations of HEP have been cross sectional studies and have not included community-based essential newborn care [6], [15]–[19]. Using cross-sectional data collected in December 2008, we reported on the association between HEP outreach activities and maternal healthcare seeking behaviors [16]. Here we report an analysis of the effectiveness of the HEP to improve maternal and newborn health care knowledge and practices at scale, using data from baseline and follow-up surveys conducted in December 2008 and December 2010. Note that the study was not designed to assess effects on measures of newborn health.

## Methods

**Figure 2:**
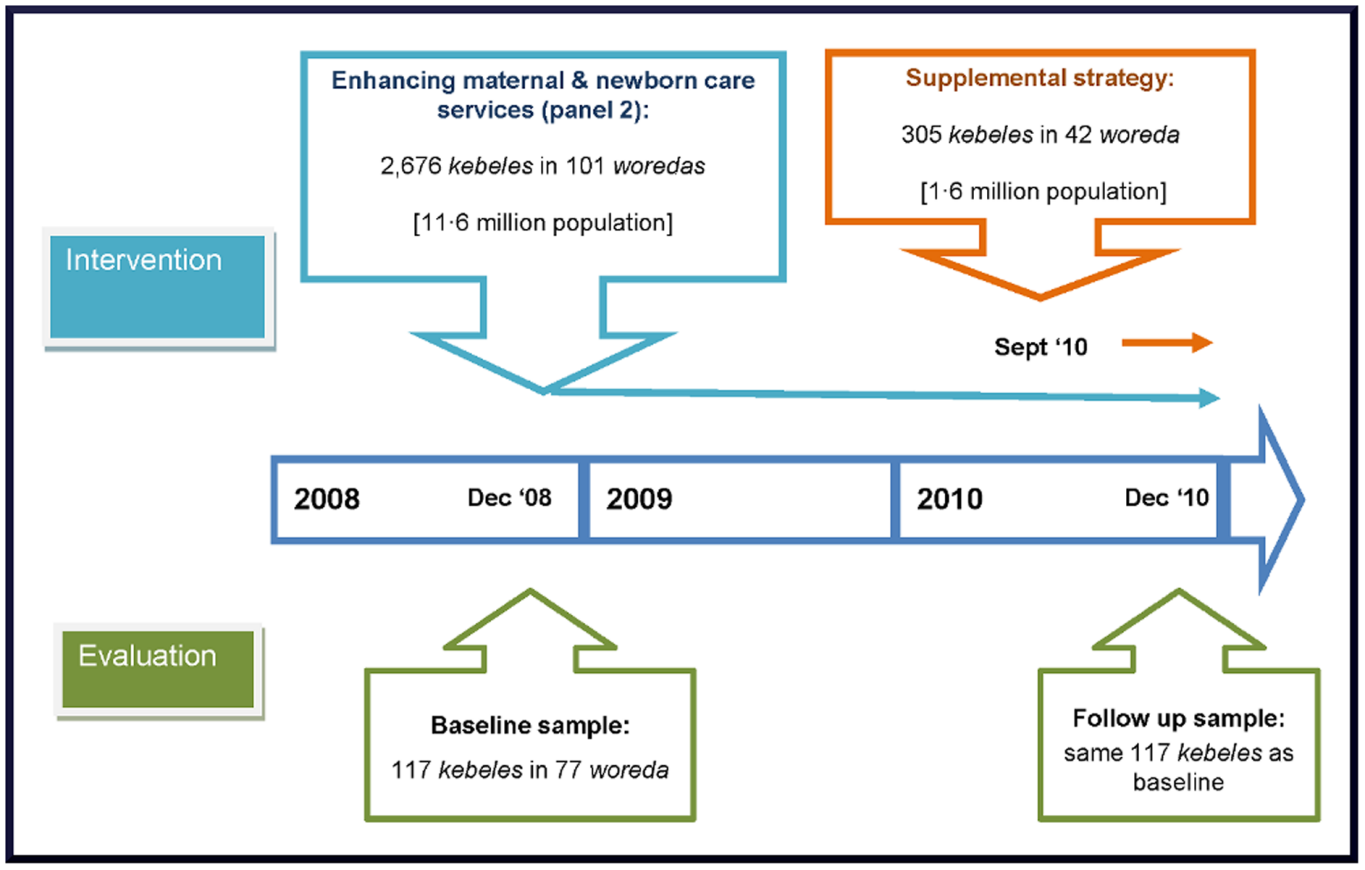
Time line for the study.

Using a plausibility design based on before-and-after surveys, we explored a dose-response relationship between the changes in program intensity measures in 117 *kebeles* between baseline and follow-up surveys and the changes in household maternal and newborn care knowledge and practices during the same period (Figure 2). The expectation was that increased program intensity will be associated with improved maternal and newborn health outcomes. We therefore used an internal comparison group, namely *kebeles* with relatively low program intensity, which were compared with those with relatively high program intensity.

### Program Description

The HEWs, young local women with high school education, were recruited by *kebele* and *woreda* councils and given one year of pre-service training [6], [8]. The Ethiopian public health system includes primary health care units (a health centre with five satellite health posts), with primary hospitals, general hospitals, and specialized referral hospitals for populations of 25,000, 100,000, 1,000,000, and 5,000,000, respectively [8],[20]. Administrative, logistical, technical, and referral support to the HEWs and the health post were provided by health centers, staffed by nurses and health officers and providing a range of basic curative services including basic emergency obstetric and neonatal care as well as primary care for maternal, neonatal, and child health [8], [21]. The context and evolution of the HEP since 2009 is shown in Table 1.

**Table 1:** Ethiopia’s Health Extension Program in context of maternal and newborn health: from 2009 to 2011.

Administrative Area	Facility, planned service and staffing
*Woreda* (100,000) α	**Primary Hospital**
	Provides: Comprehensive emergency obstetric and newborn care and other referral services
	Staffing: Medical Officers, Health Officers and Nurses
Sub-*woreda* (25,000)	**Health Centre (3,300)**β
	Provides: Curative services, administrative and technical support for all services provided by health posts, and basic emergency obstetric and newborn care
	One health centre from each *woreda* will be equipped to provide comprehensive emergency obstetric and newborn care
	Rural areas: source for primary health care for MNCH
	**Added in 2012: Basic emergency obstetric and newborn care**
*Kebele* (5,000)	**Health Post (15,000)**
	Staffing: Two health extension workers (HEWs)
	**Health Extension Worker (34,000+)**
	Requirements: female, over 18 years, 10^th^ grade education, serve communities in which they reside, one year training
	Provide: Prevention services including bed nets, sanitation, breastfeeding, safe and clean delivery, basic ANC and PNC, immunization of children and mothers, family planning and management of childhood illnesses (malaria, diarrhea, pneumonia case management)
	Seventy-five percent of their time conducts outreach activities including household visits, organize communities, train ‘model families’, and community health promoters provide
	**Community Health Promoter (300,000+)**
	Support Health Extension Workers
	Provide: health education to 25 to 30 households
	**Added in 2011: Health Development Army members (1,500,000+)**
	Replace Community Health Promoters
	Provide: health education to five households

α The average population size of the administrative area is given in parenthesis.

β Number of facilities in the country are given in parenthesis.

**Figure 3:**
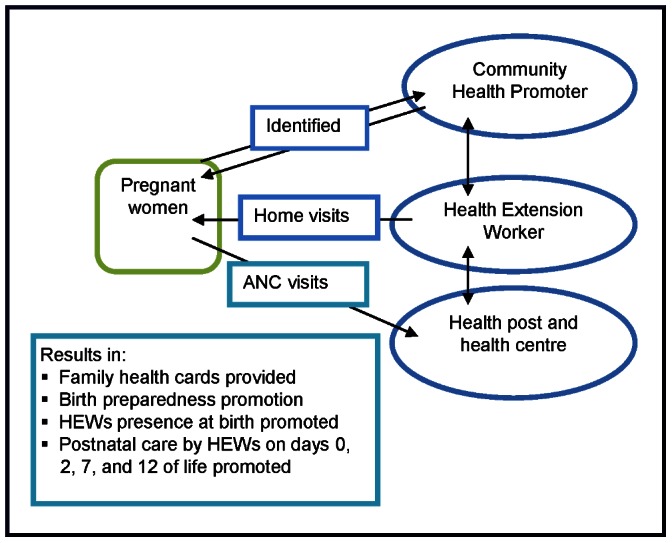
Maternal and newborn health care services provided through the HEP.

From December 2008, the L10K project supported the HEP through 12 local partner organizations. In 101 *woredas*, L10K trained and supported 5,276 HEWs to work with their communities and to organize, train, and support about 106,000 volunteer community health promoters (CHPs) from ‘model family’ households who provide maternal and newborn health services (Table 2 and Figure 3). Prior to 2011 the HEWs mainly educate and demonstrate families on hygiene and environmental sanitation (excreta disposal, solid and liquid waste management, safe water supply, food hygiene and safety, health home environment, Arthropods and rodent control, and personal hygiene), family health (Maternal and child health, reproductive health, immunization, and nutrition), disease prevention and control (HIV/AIDS, tuberculosis, malaria, and first aid) [22]. Now the ‘model family’ training include more in-depth information on maternal, newborn and child health care practices [8], [23]. Families or households that adopted 75% of the healthy practices are said to ‘graduate’ as a ‘model family’ household.

**Table 2:** Maternal and newborn health services provided by the Health Extension Program.

**1) Identification of pregnant women by Community Health Promoters (CHPs) through informal networking, then linking them with Health Extension Workers (HEWs).**
**2) Provision of Family Health Card (FHC) to pregnant women during HEW/CHP household visits, or at facility-based ANC.**
**3) Antenatal care (ANC) by the HEW at the health post)**
3.1) Encourage pregnant women to make at least four ANC facility visits and at least one visit to a health centre for review by a nurse or a health officer, and for testing urine for albumin;
3.2) Biomedical interventions: two doses or one booster of tetanus toxoid injection; iron supplementation; screening for hypertension;
3.3) Advice on nutrition during pregnancy, birth preparedness, child nutrition, immunization, and essential newborn care;
3.4) Provision of malaria prophylaxis and promotion of bed nets (malarious areas only).
**4) Promote birth preparedness through HEW/CHP household visits**
4.1) Identify and arrange for a birth attendant;
4.2) Plan for a specific birth place;
4.3) Prepare clean and appropriate materials for birth at home;
4.4 Identify a health facility for birth and emergencies;
4.5) Financial planning for childbirth and for obstetric emergencies;
4.6) Identify transport for obstetric emergencies and for birth;
4.7) Identify a suitable blood donor.
**5) Promote essential newborn care through HEW/CHP household visits**
5.1) Identify someone other than the birth attendant to take care of the newborn immediately after birth;
5.2) Encourage thermal care, cord care and immediate and exclusive breastfeeding;
5.3) Monitor the newborn for danger signs that need referral care.
**6) Promote the presence of HEW at all home births to:**
6.1) Reinforce essential newborn care messages and practices, and ensure clean delivery;
6.2) Identify danger signs for referral of mother and newborn, using a clinical algorithm;
6.3) Promote care seeking for newborn and maternal danger signs;
6.4) Provide counseling on breastfeeding and immunization.
**7) Promote postnatal care by HEWs on the first, third, 7^th^ and 12^th^ day of life to:**
7.1) Reinforce essential newborn care messages;
7.2) Provide breastfeeding counseling;
7.3) Check for newborn and postpartum illnesses, and refer if necessary, using a clinical algorithm.

The CHPs used a Family Health Card (FHC), a booklet with pictorial messages, to promote focused antenatal care; birth preparedness measures; clean and safe childbirth; recognition of danger signs needing referral in pregnancy, childbirth, and the postnatal period; essential newborn care; infant and childhood nutrition, immunization, and danger signs of childhood illnesses; and household hygiene and sanitation measures (the FHC can be accessed from www.l10k.jsi.com/Resources/FHC-Eng.pdf). The L10K project implemented supplemental community-based strategies including participatory community quality improvement, a community solutions fund, and non-financial incentives for CHPs in 42 *woredas* from September 2010. These strategies had negligible implications for this study because the data was collected in December 2010, by which time very few births in the previous year could have been affected by the supplemental strategies (Figure 2).

### Data Collection

Two-stage stratified cluster sampling was done to obtain family planning information from women aged 15 to 49 years; maternal, newborn, and infant health and nutrition information from women with children 0 to 11 months; and child immunization and childhood illness information from women with children 12 to 23 months. The survey instruments for the three target groups were adapted from Demographic and Health Survey [2] and Saving Newborn Lives questionnaires, and then translated into the three major local languages (Amharic, Oromifa, and Tigregna). In Southern Nations and Nationalities People’s Region (SNNPR), with 11 more languages, the interviewers translated from Amharic while administering the questionnaires. Ethical clearance was obtained from the Ethiopian Public Health Association. Verbal consent was sought and documented by the interviewer. If the respondent was less than 18 years old then consent was sought from her husband or guardian. Majority of the respondents were not expected to be able to read or write; as such, written consent was not sought. If the respondent agreed to be interviewed after listening to the consent statement the interviewer marked the questionnaire as consent given below the consent statement and then signed below that. The interviewer continued with the interview only after receiving and documenting the consent. The survey protocol submitted to the Ethiopian Public Health Association's ethical review committee included the study questionnaire with the consent statement. The protocol also described the consent obtaining procedure which was approved by the committee. The name and address of the respondent was not recorded by the interviewer. As such, the study database contained the records of anonymous respondents which was analyzed for this study.

At the first stage, *kebeles* were selected as clusters with probability proportional to their estimated population sizes, and using implicit stratification by region. At the second stage, the 30 by seven cluster survey strategy was used to obtain information from the three target respondents [24]. In brief, the first household was selected from the middle of the *kebele* and then every fifth household was visited and all consenting women aged 15–49 years were interviewed. From each *kebele,* a quota of 20 interviews with women aged 15–49 years, 12 women with children 0 to 11 months, and ten women with children 12–23 months was set during the baseline survey, and a quota of 12 respondents from each of the three target groups was set for the follow-up survey. After reaching the quota for women aged 15–49 years in a *kebele* the interviewers only sought to conduct interviews for the other target groups.

The interviewers and supervisors were health professionals from regional health bureaus, who received five days of training, including a day of field practice. They did not interview in the areas under their supervision. Survey supervisors and regional coordinators were trained to monitor and supervise the work and ensure data quality. Each survey, including the training period, took about a month. Data was entered twice and differences resolved with reference to the original forms.

### Program Intensity Measurements

The HEP intensity was estimated through household members’ reported exposure to the program. To avoid individual-level selection bias, caused for example by, health-conscious individuals choosing to participate in the program, intervention bias caused by providers targeting individuals based on health behavior, and recall bias caused by differential recall of exposure based on health behavior, we used different respondent groups for measuring program exposure and outcomes. The HEP intensity measures were *kebele*-level averages obtained from exposure to the HEP reported by women of reproductive age and women with children 12 to 23 months old. The *kebele*-level HEP intensity measure excluded women with children 0 to 11 months among whom the outcomes of interest were measured.

The *kebele*-level measures of HEP intensity were based on outreach activities of the HEWs: 1) the *period prevalence of household visits by HEWs*, defined as the percentage of women in a *kebele* who were visited by a HEW during six months preceding the survey; 2) the *period prevalence of household visits by CHPs*, defined as the percentage of women in a *kebele* who were visited by a CHP during the last six months; 3) the *proportion of households with a FHC*; and 4) the *proportion of model families*, defined as the percentage of respondents who reported that their household was a *model family* household or they were working towards it.

A *program intensity score* was given to each *kebele* by summing the four HEP intensity items with equal weight. The score was recalibrated to range between zero and ten, with a higher score indicating better performance. Cronbach’s alphas were calculated to assess the internal reliability of the four items in measuring the underlying construct of program intensity. The possible values of alpha ranges between zero and one, and values exceeding 0.70 are regarded acceptable [25]. The Cronbach’s alpha for the four items was 0.77. Item analysis indicated all the four items were required to have the maximum reliability [26].

### Outcome Measures

The essential maternal and newborn care practices that were expected to contribute towards improved neonatal survival [5] included 1) antenatal care, including birth preparedness, 2) safe childbirth and postnatal care, 3) essential newborn care, and 4) women’s knowledge about danger signs. Outcome measures are described in Table 3.

**Table 3:** Maternal and newborn health care knowledge and practice indicators.

**1) Antenatal care and birth preparedness**		
1.1) Receiving any antenatal care (ANC) service at a health facility;		
1.2) Iron supplementation at least once during ANC;		
1.3) At least two tetanus toxoid injections during ANC;		
1.4) The number of preparedness measures taken, including financial, transport, food, birth attendants, identifying a health facility, preparing clean materials for delivery, identifying a blood donor.		
**2) Safe childbirth and postnatal care**		
2.1) Institutional delivery at a health centre or hospital;		
2.2) Delivery assisted by skilled birth attendant (doctor, nurse, or a midwife);		
2.3) Receiving post-natal care at home by a Health Extension Worker (HEW);		
2.4) Receiving post-natal care by a HEW within seven days of childbirth.		
**3) Essential newborn care – thermal care** :		
3.1) Newborn is dried and wrapped immediately following childbirth (or within an hour);		
3.2) Bathing the newborn is delayed by more than six hours;		
3.3) Skin-to-skin contact with the newborn always–as opposed to often, few times, or never maintained;		
3.4) Took thermal care: dried and wrapped baby, delayed bathing, and maintained skin-to-skin contact.		
**4) Essential newborn care – clean cord care:**		
4.1) Cut umbilical cord with sterile instrument among deliveries without skilled attendance;		
4.2) Tying the umbilical cord with sterile thread among deliveries without skilled attendance;		
4.3) Apply nothing on the cut stump of the umbilical cord;		
4.4) Clean cord care: if the umbilical cord was cleanly cut and tied, and nothing was applied to the stump, among deliveries without a skilled attendant.		
**5) Essential newborn care – breastfeeding**:		
5.1) Newborn is given colostrum (first milk);		
5.2) Newborn is put to breast immediately (within one hour) of birth;		
5.3) Exclusively breast feeding the baby during the last 24 hours (among women with a child aged less than one month).		
**6) Knowledge of maternal and newborn danger signs**: **Unprompted knowledge variables of 11 childbirth, five postpartum and 11 neonatal danger signs** α		
**Childbirth danger signs**	**Postpartum maternal danger signs**	**Neonatal danger signs**
Excessive vaginal bleeding	Excessive vaginal bleeding	Vomiting
Foul-smelling discharge	Foul-smelling discharge	Fever
High fever	High fever	Poor sucking or feeding
Baby’s hand or feet come first,	Severe abdominal pain	Difficulty in breathing,
Baby in abnormal position	Convulsions	Baby feels cold
Prolonged labor >12 hours		Baby too small/early birth
Retained placenta		Redness/discharge on cord
Ruptured uterus		Red swollen eye/discharge,
Prolapsed cord		Yellow palm/sole/eye,
Cord around neck,		Lethargy
Convulsions		Unconscious

α Cronbach’s coefficients of the three knowledge scores were low (<0•37). The knowledge scores reflected the number of correct knowledge items spontaneously recalled by women.

### Statistical Analysis

The *kebele*-level confounders–i.e., program placement bias–were the greatest threat to the validity of the dose-response-analysis. First, a graphical analysis was done to visualize the possible associations between program exposure and the outcomes. The analysis plotted the *kebele-*level difference in the prevalence of a maternal and newborn care between the survey periods on the y-axis against the *kebele*-level difference in *program intensity score* on the x-axis with an ordinary least square (OLS) line of the scatter plot, i.e., a fitted line, drawn to inspect the possible dose-response associations. The fitted line can be explained by the following equation:
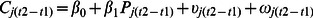
(1)


The average change in the prevalence of a maternal and newborn care practice ‘*C*’ in *kebele* ‘*j*’ between baseline ‘*t1*’ and follow-up ‘*t2*’ is denoted by ‘*C_j(t2-t1)_*’; changes in *program intensity score* ‘*P*’ between baseline ‘*t1*’ and follow-up ‘*t2*’ in kebele ‘*j*’ is denoted by ‘*P_j(t2– t1)_*’; ‘*υ_j(t2– t1)_*’ is *kebele*-level residuals or unexplained variances (including confounders and other explanatory factors) that are fixed over time; and ‘*ω_j(t2– t1)_*’ denotes *kebele*-level residuals or the unexplained variance of the outcome which change over time. The value of ‘*υ_j(t2– t1)_*’ is zero because the residuals that are similar between the survey periods are differenced out; ‘*β_1_*’ measures the program effect; and ‘*β_0_*’ measures the changes in the outcome between the survey periods that is not explained by ‘*P_j(t2– t1)_*’.

The program effect, i.e., ‘*β_1_*’ estimated by Equation 1 is prone to ecological bias [27]. For example, the *kebele*-level ecological association between exposure and outcome considers that all individuals within a *kebele* are homogenous in response to program exposure. The assumption is not reasonable because program uptake would likely to be different according to the differentials in education and other background characteristics of the individuals in the *kebele*.

In such cases, the multi-level analysis is appropriate which allows assessing the associations between *kebele*-level contextual measures of program intensity and individual-level maternal and newborn care behavioral outcomes, net of the individual-level background characteristics [27]. The multi-level model of choice was the *kebele*-level fixed effect as opposed to the *kebele*-level random-effects. Although the latter model is more efficient than the former, the estimates from the latter are sensitive to inaccuracies [28]–[30], which would likely occur in one or more of the models that were estimated for this paper. The multi-level model also accounted for cluster-survey design effect (i.e., the intra-class correlation within clusters) [30]. Equation 2 describes the *kebele*-level fixed effects model.
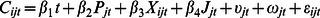
(2)


A maternal and newborn care practice ‘*C*’ among individuals ‘*i*’ nested within *kebele* ‘*j*’ during survey period ‘*t*’ is denoted by ‘*C_ijt_*’; the *program intensity score* ‘*P*’ in *kebele* ‘*j*’ during survey period ‘*t*’ is denoted by ‘*P_jt_*’; the vector of measured household and respondent characteristics among the individuals ‘*i*’ nested within *kebele* ‘*j*’ during survey period ‘*t*’ is denoted by ‘*X_ijt_*’; the vector of measured *kebele*-level contextual factors are denoted by ‘*J_jt_*’ (the individual, household and *kebele*-level factors–i.e., ‘*X*’ and ‘*J*’–are listed in Table 4); the secular trend is captured by ‘*t*’ denoting the survey period; *kebele-*level residuals that do not change over time (i.e., fixed over time) is ‘*υ_jt_*’, which like the value of ‘*υ_j(t2– t1)_*’ in Equation 1, is zero; ‘*ω_jt_*’ is the time varying *kebele*-level residuals (i.e, unexplained factors); and, ‘*ε_ijt_*’ is the individual-level residuals. The parameter of interest is ‘*β_2_*’, i.e., the program effect. Missing values for the women and household characteristics were replaced with non-missing responses for that variable during the same period, randomly obtained from respondents with similar background characteristics [31]. Using Stata 12.1, the logit and the ordinary least square versions of the *kebele*-level fixed-effects regression model was estimated for binary (for care practices) and continuous outcomes (for knowledge scores), respectively.

**Table 4:** Characteristics of the respondents: women with children 0 to 11 months, baseline and follow-up surveys.

Respondent characteristics		Baseline		Follow-up		p-value
		%	N	%	N	
*All women*		100•0	1,404	100•0	1,404	
*Women's age (years)*	15–19	8·7	122	8·3	116	0•114
	20–34	76·4	1,073	73·7	1,035	
	35–49	14·3	201	17·3	243	
	Missing	0·6	8	0·7	10	
Age of last child (months)	<1	5·3	74	4·6	64	0•003
	1–5	40·1	563	47·3	664	
	6+	54·2	761	47·7	670	
	Missing	0·4	6	0·4	6	
Marital status	Unmarried/single	5·0	70	5·3	75	0•699
	Married/living together	94·4	1,325	93·8	1,317	
	Missing	0·6	9	0·9	12	
Education	None	75·5	1,060	75·4	1,059	0•194
	Primary	14·0	196	13·5	190	
	Secondary or higher	7·3	102	9·3	131	
	Missing	3·3	46	1·7	24	
Number of children	1	19·0	266	18·9	265	0•424
	2	18·7	263	16·3	229	
	3	16·5	232	17·0	239	
	4+	45·2	634	47·2	663	
	Missing	0·6	9	0·6	8	
Religion	Orthodox	59·1	830	58·3	819	0•347
	Protestant	13·3	186	15·2	213	
	Muslim	25·6	359	24·6	345	
	Traditional/other	1·4	19	1·0	14	
	Missing	0·7	10	0·9	13	
Distance to drinking water source	In compound	3·4	47	2·1	30	<0•001
	<30 minutes	74·8	1,050	85·7	1,203	
	30+ minutes	21·0	295	11·8	165	
	Missing	0·9	12	0·4	6	
Distance to any health facility	<30 min.	53·1	745	62·0	871	<0•001
	30 min–<1 hr	24·1	338	28·3	397	
	1-<2 hrs	14·8	208	7·5	105	
	2+ hrs	7·5	105	1·8	25	
	Missing	0·6	8	0·4	6	
Frequency of listening to radio	Almost every day	20·3	285	21·7	304	0•137
	At least once a week	13·8	194	13·0	183	
	Less than once a week	4·6	65	3·0	42	
	Not at all	60·5	849	61·7	866	
	Missing	0·8	11	0·6	9	
Wealth quintile β	Poorest	20·7	291	20·1	282	0•274
	Medium poor	20·7	290	21·2	298	
	Middle	21·5	302	20·3	285	
	Medium rich	22·7	318	20·8	292	
	Richest	13·4	188	16·6	233	
	Missing	1·1	15	1·0	14	
Distance to basic emergency	1	21·4	300	17·1	240	0•184
obstetric care from *kebele* (hours)	2	20·5	288	17·1	240	
	3	10·3	144	20·5	288	
	4+	42·7	600	42·7	600	
	Missing	5·1	72	2·6	36	

β The wealth index score was constructed for each household with the principal component analysis of the household possessions (electricity, watch, radio, television, mobile phone, telephone, refrigerator, table, chair, bed, electric stove, and kerosene lamp), and household characteristics (type of latrine and water source). The households were ranked according to the wealth score and then divided into five quintiles indicating poor, medium poor, medium, medium rich and rich households [32].

The likelihood ratio global statistics of the logit models and the global F-statistics of the linear regression models were used to assess the goodness-of-fit of the models.

The fixed-effect model applied to panel surveys with two points in time (such as our study design) is analogous to the first difference model described by Equation 1 [28]. As such, ‘*β_2_*’ of Equation 2 can also be interpreted as the effect of the *kebele*-level changes in program intensity on individual-level maternal and newborn care outcome.

Lastly, a counterfactual analysis was done to quantify the program effects on maternal and newborn care practices. First, we predicted the prevalence of a maternal and newborn care practice by using the multi-level model described by Equation 2. Then we replaced the value of *program intensity score* with zero to estimate counterfactual prevalence of that maternal and newborn care behaviour. This counterfactual prevalence simulates what would have happened if the HEP outreach activities did not take place. The difference between the actual prevalence and the counterfactual prevalence provides an estimate of the change in the maternal and newborn care practice attributable to the HEP outreach activities. Only the statistically significant program effects were simulated and the fraction (or mean) attributable was reported. Effects of the constituent items of the *program intensity score* were also estimated.

**Data availability**: The data together with the Stata syntax files are available from the corresponding author on request.

## Results

The 117 *kebeles* were from 77 of the 101 intervention *woredas* and included 3,556 and 3,502 women respondents during the baseline and follow-up surveys, respectively, among which were 2,340 women aged 15–49 years, 1,404 women with children 0 to 11 months, and 1,170 women with children 12 to 23 months during the baseline survey and 1,404 women from each of these three target groups during the follow-up survey. The average number of respondents per *kebele* for the program intensity measures was 18 during both the survey periods.

### Respondent Characteristics

The distribution of women’s age, marital status, education, parity, religion, frequency of radio listenership, household wealth quintile, and the distance to basic emergency obstetric care from the *kebele* were similar in the two surveys (Table 4). There was some evidence of a change in the age of the youngest child (54% were over 6 months old in the 2008 sample, compared to 48% in 2010), in women living more than 30 minutes from the source of drinking water (from 21% to 12%), and in women living over an hour away from any health facility (from 22% to 9%).

### Program Intensity

The *program intensity score* almost doubled, increasing from 2.2 at baseline to 4.0 during follow-up (Table 5). All four constituent items improved–the prevalence of household visits by HEWs, household visits by CHPs, possession of FHC, and households with ‘model families’ in a *kebele* increased by a factor of 1.3, 1.6, 3.9, and 2.5, respectively.

**Table 5:** *Kebele*-level HEP intensity measures at baseline and follow-up surveys (n = 117).

Program intensity measures		Baseline	Follow-up	Change	(95% CI)
Household visits by a HEW	Mean	37·0	49·2	12·3	(6·2–18·3 )
	Median	40·0	50·0	10·0	(1·4–18·6)
Household visits by a CHP	Mean	20·6	33·3	12·6	(8·5–16·8)
	Median	13·0	33·3	20·3	(11·7–28·9)
FHC possession	Mean	8·6	33·7	25·1	(20·7–29·5)
	Median	0·0	30·0	30·0	(23·2–36·8)
‘Model family’ household	Mean	11·0	27·6	16·6	(12·4–20·8)
	Median	6·7	22·7	16·1	(9·3–22·8)
***Program intensity score***	Mean	2·2	4·0	1·9	(1·5–2·2)
	Median	2·0	4·1	2·1	(1·6–2·6)

### Maternal and Newborn Health Care Practices

With the exception of tetanus toxoid injection during pregnancy and cutting the umbilical cord with a sterile instrument, we found evidence of improvement in all maternal and newborn care practices (Table 6). The improvements were over ten percentage points for at least one antenatal visit, iron supplementation, receiving any post-natal care, delay in bathing the newborn, thermal care of the newborn, and exclusive breastfeeding; and between five and ten percentage points for taking any birth preparedness measure, receiving post-natal care within seven days of childbirth, drying and wrapping the baby immediately after birth, maintaining skin-to-skin contact, clean cord care, giving colostrum, and breastfeeding immediately after childbirth. Smaller improvements, of less than five percentage point were seen in: tetanus toxoid injection, institutional deliveries, home deliveries assisted by skilled birth attendants, tying the cord with sterile thread, and applying nothing to the cut cord. There were improvements in three scores of women’s knowledge of danger signs.

**Table 6:** Changes in maternal and newborn health indicators between baseline and follow-up surveys.

Maternal & newborn care outcomes	Baseline		Follow-up		Change	
Prenatal care and birth preparedness	Estimate	Obs.	Estimate	Obs.	Estimate	(95% CI)
% received antenatal care (ANC)	57·6	1,401	70·2	1,403	12·6	(9·5–15·8)
% given iron supplement	13·7	1,401	31·7	1,403	18·0	(15·3–20·8)
% received at least two TT injection	41·3	1,389	43·6	1,392	2·4	(–0·1–5·9)
% taken any birth preparedness measures	69·4	1,400	75·1	1,395	5·6	(2·4–8·9)
**Safe delivery and postnatal care**						
% had institutional delivery	6·2	1,398	10·6	1,398	4·4	(2·6–6·3)
% of deliveries assisted by skilled birth attendance	8·2	1,404	11·3	1,404	3·1	(1·0–5·1)
% received PNC	4·6	1,404	15·3	1,404	10·8	(8·6–12·9)
% received PNC in seven days	3·1	1,404	9·9	1,404	6·8	(5·0–8·6)
**Essential newborn care**						
Thermal care						
% dried and wrapped baby immediately following childbirth	69·4	1,404	75·0	1,404	5·6	(2·4–8·8)
% delayed bathing the newborn by more than six hours	25·3	1,365	38·4	1,373	13·1	(10·0–16·3)
% always maintained skin-to-skin contact with newborn	70·8	1,403	76·9	1,400	6·1	(3·2–9·0)
% took thermal care	10·5	1,364	24·5	1,372	14·1	(11·4–16·8)
Clean cord care						
% of home deliveries cut umbilical cord with sterile instrument	95·9	1,233	96·0	1,163	0·2	(–1·3–1·8)
% of home deliveries tied umbilical cord with sterile thread	57·6	1,233	62·4	1,163	4·8	(0·9–8·8)
% applied nothing on umbilical cord cut	67·7	1,348	74·0	1,338	4·3	(1·1–7·3)
% took clean cord care	36·3	1,190	45·6	1,109	9·3	(5·3–13·3)
Breastfeeding practices						
% gave baby colostrums	46·2	1,404	53·3	1,404	7·1	(3·5–10·6)
% put baby to breast immediately after birth	46·0	1,404	54·2	1,404	8·2	(4·7–11·6)
% exclusively breastfeeding their neonates	82·4	74	95·3	64	12·9	(2·2–23·5)
**Knowledge scores(means)**						
Maternal danger sign during childbirth score (0–11)	1·90	1,396	2·20	1,400	0·30	(0·22–0·38)
Maternal danger sign during postnatal period score (0–5)	1·39	1,388	1·70	1,397	0·31	(0·25–0·38)
Neonatal danger sign score (0–11)	1·87	1,400	2·03	1,397	0·26	(0·10–0·24)

### Association between Program Intensity and Maternal and Newborn Health Care Practices

**Figure 4:**
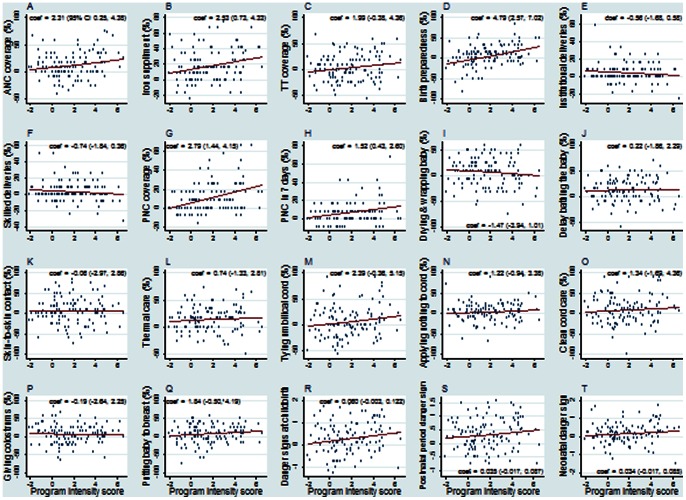
Kebele-level correlation between HEP intensity and maternal and newborn outcomes. Kebele-level scatter plots with fitted regression lines between changes in maternal and newborn health care practices/knowledge and changes in *program intensity score* between 2008 and 2010 (n = 117). Foot note: Regression coefficient of the fitted line and its 95% confidence interval are included with the figures.

An increase in *program intensity score* at the *kebele-*level was associated (p<0.05) with improvements in antenatal care, iron supplementation, taking any birth preparedness measure, receiving any postnatal care, and receiving postnatal care in 7 days (Figure 4). For example, every one unity (i.e., 10 percent-points) change in *program intensity score* the proportion of mothers who received antenatal care increased by 2.3 percentage-points.

Estimated effects of *kebele*-level HEP intensity measures on household maternal and newborn care practices and knowledge scores were obtained from *kebele*-level fixed-effects logit and linear regression models, respectively, adjusted for secular trend and respondent, household and *kebele* characteristics (Table 7).

**Table 7:** Adjusted effects of *kebele*-level HEP intensity measures on maternal and newborn health indicators.

Maternal & newborn care outcomes	HH visits by HEW			HH visits by CHP			FHC possession			Model family HH			*Program intensity score*		
***Prenatal care and birth preparedness***	OR	(95% CI)	p-value	OR	(95% CI)	p-value	OR	(95% CI)	p-value	OR	(95% CI)	p-value	OR	(95% CI)	p-value
Received antenatal care (ANC)	1·06	(1·00–1·12)	0•047	1·11	(1·02–1·20)	0•011	1·05	(0·97–1·13)	0•220	1·08	(1·00–1·17)	0•054	1·13	(1·03–1·23)	0•008
Given iron supplement	1·01	(0·94–1·09)	0•719	1·13	(1·03–1·24)	0•008	1·16	(1·07–1·27)	0•003	1·04	(0·94–1·14)	0•474	1·14	(1·02–1·26)	0•018
Received at least two TT injection	1·06	(1·01–1·12)	0•020	1·11	(1·03–1·20)	0•004	1·04	(0·97–1·11)	0•293	0·98	(0·91–1·05)	0•508	1·09	(1·00–1·18)	0•043
Taken any birth preparedness measures	1·16	(1·10–1·23)	<0•001	1·17	(1·07–1·28)	<0•001	1·16	(1·07–1·26)	0•001	1·20	(1·10–1·30)	<0•001	1·31	(1·19–1·44)	<0•001
***Safe delivery and postnatal care***															
Had institutional delivery	0·92	(0·83–1·01)	0•092	0·97	(0·83–1·13)	0•718	0·98	(0·86–1·12)	0•782	0·90	(0·77–1·06)	0•203	0·89	(0·75–1·05)	0•164
Skilled birth attendance	0·94	(0·86–1·03)	0•194	0·94	(0·82–1·08)	0•380	0·95	(0·84–1·07)	0•392	0·87	(0·75–1·00)	0•054	0·87	(0·75–1·02)	0•082
Received any PNC	1·35	(1·21–1·51)	<0•001	1·39	(1·20–1·61)	<0•001	1·22	(1·07–1·40)	0•003	1·20	(1·01–1·42)	0•036	1·60	(1·34–1·91)	<0•001
Received PNC within seven days	1·25	(1·10–1·42)	<0•001	1·36	(1·14–1·61)	0•001	1·24	(1·06–1·45)	0•007	1·20	(0·98–1·45)	0•071	1·53	(1·24–1·88)	<0•001
***Essential newborn care***															
Dried and wrapped baby immediately following birth	1·00	(0·95–1·06)	0•865	0·98	(0·91–1·06)	0•675	0·94	(0·87–1·02)	0•119	0·87	(0·80–0·93)	<0•001	0·93	(0·85–1·01)	0•085
Delayed bathing the newborn by more than six hrs.	0·99	(0·93–1·05)	0•749	1·00	(0·91–1·08)	0•915	1·10	(1·01–1·19)	0•024	0·90	(0·82–0·98)	0•011	0·99	(0·90–1·09)	0•835
Always maintained skin-to-skin contact with newborn	1·10	(1·03–1·18)	0•004	1·00	(0·91–1·09)	0•940	1·12	(1·03–1·23)	0•010	0·83	(0·77–0·90)	<0•001	1·02	(0·93–1·13)	0•646
Took thermal care of baby	1·07	(1·00–1·16)	0•066	1·02	(0·92–1·13)	0•691	1·13	(1·02–1·24)	0•021	0·76	(0·69–0·85)	<0•001	1·00	(0·89–1·12)	0•983
Tied umbilical cord with sterile thread	1·09	(1·02–1·16)	0•013	1·21	(1·10–1·34)	<0•001	1·04	(0·95–1·14)	0•407	1·04	(0·95–1·14)	0•363	1·15	(1·04–1·27)	0•006
Applied nothing on the cut umbilical cord	1·05	(0·99–1·11)	0•115	0·99	(0·91–1·08)	0•824	1·02	(0·95–1·10)	0•606	1·03	(0·94–1·11)	0•552	1·05	(0·96–1·15)	0•320
Took clean cord care	1·04	(0·98–1·10)	0•192	1·10	(1·01–1·20)	0•037	1·00	(0·92–1·08)	0•978	0·99	(0·91–1·08)	0•901	1·06	(0·96–1·16)	0•245
*Breastfeeding practices*															
Gave baby colostrums	1·00	(0·96–1·06)	0•777	0·94	(0·88–1·02)	0•122	1·01	(0·95–1·09)	0•673	1·01	(0·94–1·08)	0•756	1·00	(0·92–1·08)	0•915
Putting baby to breast immediately after birth	1·04	(0·99–1·10)	0•128	1·00	(0·93–1·08)	0•974	1·12	(1·05–1·21)	0•001	1·08	(1·00–1·16)	0•048	1·10	(1·01–1·20)	0•017
***Knowledge scores***	Coef.			Coef.			Coef.			Coef.			Coef.		
*Maternal danger sign during childbirth (0–11)*	0·02	(−0·01,0·04)	0•143	0·05	(0·02, 0·09)	0•004	0·04	(0·00, 0·07)	0•044	0·05	(0·01, 0·09)	0•007	0·06	(0·02, 0·10)	0•002
*Maternal danger sign during postnatal period (0–5)*	0·01	(−0·01,0·03)	0•590	0·05	(0·02, 0·08)	0•001	0·03	(0·00,0·06)	0•032	0·01	(−0·02,0·04)	0•512	0·04	(0·00,0·07)	0•031
*Neonatal danger sign (0–11)*	0·00	(−0·02,0·02)	0•858	0·04	(0·01, 0·07)	0•009	0·04	(0·01, 0·07)	0•006	0·03	(−0·01,0·06)	0•114	0·04	(0·00,0·07)	0•034

For a ten percentage point increase in the *program intensity score,* the odds of a woman having received antenatal care increased by 13%; the odds of iron supplementation increased 14%; the odds of receiving at least two tetanus toxoid injections increased by 9%; the odds of taking any birth preparedness measure increased by 31%; the odds of receiving any postnatal care by a HEW increased by 60%; the odds of receiving postnatal care by a HEW within seven days of childbirth increased by 53%; the odds of tying the umbilical cord with sterile or clean thread increased by 15%; the odds of putting baby to breast immediately after childbirth increased by 10%; and the average numbers of correct responses recalled for danger signs during childbirth, during postnatal period, and during neonatal period increased respectively by 0.06, 0.04, and 0.04.

Although we found no association between *program intensity score* and delayed bathing, maintaining skin-to-skin contact with the newborn, or taking thermal care of the newborn, there were associations at *kebele* level between these practices and the proportion of households having FHCs. Similarly, *kebeles* with larger increases in the prevalence of HEW visits had higher proportions of mothers maintaining skin-to-skin contact.

Contrary to expectation, we found some evidence that *kebeles* with increases in the prevalence of ‘model family’ households had a *decrease* in thermal care of the newborn.

We found no evidence that the *program intensity score* or its constituent items were associated with institutional deliveries, deliveries assisted by health professionals, applying nothing to the cord, and giving the baby colostrum.

The counterfactual analysis indicated that the program effects, i.e., the effects of *program intensity score* on maternal and newborn care practices ranged between seven (TT vaccination) and 20 (for birth preparedness) percentage points (Table 8), The effect of the *program intensity score* on increasing the women’s mean knowledge scores increased between 0.14 and 0.25.

**Table 8:** Counterfactual analysis of the effects of HEP intensity measures on maternal and newborn health indicators.

Maternal and newborn care outcomes	HH visits by HEW	HH visits by CHP	FHC possession	Model family HH	*Programme performance score*
***Prenatal care and birth preparedness***					
Received antenatal care (ANC)	5.2	6.5			8.9
Given iron supplement		5.9	7.7		7.4
Received at least two TT injection	6.3	7.6			7.0
Taken any birth preparedness measures	14.0	9.7	9.2	8.9	20.1
***Perinatal and postnatal care***					
Had institutional delivery					
Skilled birth attendance					
Received any PNC by HEW	8.5	7.7	5.8	4.4	11.2
Received PNC by HEW within 7 days	5.4	5.4	4.7		7.8
***Essential newborn care***					
Dried and wrapped baby				−7.3	
Delayed bathing the newborn			5.5	−5.4	
Always maintained skin-to-skin contact	8.9		7.0	−8.5	
Took thermal care			5.5	−10.6	
Tied umbilical cord with sterile thread	7.2	11.8			10.1
Applied nothing on the cut umbilical cord					
Took clean cord care		6.3			
Gave baby colostrums					
Putting baby to breast immediately after childbirth			8.5	4.3	8.4
***Knowledge scores***					
*Maternal danger sign during childbirth (0–11)* µ		0.18	0.12	0.14	0.25
*Maternal danger sign during postnatal period (0–5)* µ		0.17	0.10		0.14
*Neonatal danger sign (0–11)* µ		0.14	0.14		0.15

Only the statistically significant effects in Table 7 are reported here.

µ All the program effects are attributable fractions (percentage-points); while the program effects on knowledge are attributable means.

## Discussion

Our study is unusual in reporting effectiveness of community-based newborn survival interventions integrated within the HEP at scale, in a population of 11.6 million people. We found strong evidence of a dose-response relationship between the HEP and better care practices, which indicate that the program is an effective platform for improving community-based newborn care practices at scale. Among the four strategic elements of outreach making up our *program intensity score*, the FHC was associated with more outcomes than other elements, followed by household visits by CHP, training of ‘model families’, and household visits by HEW. A lack of appropriate comparison areas is a major challenge to large-scale effectiveness evaluations. A dose-response relationship between program intensity and the outcomes of interest allows a stronger plausibility statement than other options [33], [34], and we applied this approach in maternal and newborn program effectiveness evaluation.

We previously reported a cross-sectional association between maternal care practices and HEP outreach intensity measures observed in December 2008, before strengthening the essential newborn care practices package evaluated here [16]. The relationships between the HEP and maternal health care practices observed prospectively were similar to those at baseline–validating the baseline observation. Nevertheless Admassie et al. (2009) and Medhanyie et al. (2010) studies, which represented 10 and three districts, respectively, did not report any such evidence.

Prior to 2011 the ‘model family’ training module did not include essential newborn care practices. If a ‘model family’ household had cultural practices that were undesirable for newborn health, then by virtue of being a model in the neighborhood they would be promoting this undesirable practice. The apparent undesirable influence of ‘model families’ on thermal care of the newborn should be mitigated by the introduction of the updated ‘model family’ training module that includes essential newborn care practices [8], [23].

There are several limitations to this study. First, the findings from the *kebele*-level fixed-effect models are not generalisable outside the sample [28]. Second, the 30 by seven method can be criticized because the interviewers may avoid hard-to-reach areas and non-responders are not revisited [24]. Moreover, the outcomes considered are associated for the most recent birth among women with surviving children 0 to 11 months old, excluding women whose children died before the opportunity for an interview. It is likely that such cases would have relatively poor practices; their exclusion would therefore result in overestimating the care practices. Nevertheless, since the sampling method was consistent between the surveys, and since it is changes between baseline and follow-up that were assessed in the analysis, the *kebele*-level biases that are consistent over time do not affect the results. Third, any *kebele*-level confounders that vary over time would bias the study findings. On one hand, the attribution of effects to the HEP could be overestimated if the improvement in HEP intensity was higher in areas where there have been improvements in other developmental factors that also influenced maternal and newborn care practices. On the other hand, the program effect could be underestimated if the improvement in HEP intensity was higher in areas with relatively poor access to health services, in which case the target population in areas with lower HEP intensity would be utilizing services other than the HEP, resulting in no effect or even a negative effect in the dose-response relationship. Fourth, the exploratory analysis of this paper tested a large number of hypotheses; as such, some of the apparent program effects may be spurious. Lastly, the exposure period was not uniform because: HEW training did not start simultaneously everywhere, and also the reference period of the outcomes of interest varied between 0 to 11 months preceding the survey (and more, for interventions during pregnancy). Shorter exposure in some areas would likely result in underestimation of the program effects. Lastly, although the program exposure and program outcomes were measured independent of each other, the dose-response effects could still be subject to recall bias; which if present, would be in an unknown direction, leading to spurious relationships between program exposure and the expected behavioral outcomes. Since the *program intensity score* systematically predicted the maternal and newborn care practices in the expected direction, it is unlikely that the recall bias was critical.

Nevertheless, the validity of the scale measuring program intensity, i.e., the *program intensity score*, was reasonable, mainly because 1) on face value the four strategic elements of the scale captures key outputs that result from the outreach activities of HEP; 2) the internal reliability of the scale was reasonable (Cronbach’s alpha was 0.77); and 3) the scale predicted maternal and newborn care behaviors in the expected directions [25].

Although there was improvement over a two year period in the proportion of deliveries done in health facilities and attended by skilled health professionals; drying and wrapping the newborn immediately following childbirth; applying nothing on the cut umbilical cord; and giving colostrum, we found no evidence that the HEP outreach strategies were responsible for these changes. The improvements in these indicators could be due to other aspects of the HEP. For example, improvement in skilled deliveries could be explained by improved availability and accessibility to the service. Providing health education to mothers on newborn health care practices is generally done through antenatal visits; in which case, the intensity of the HEWs outreach activities would not be associated with them. However, implementation research will be required to identify why HEP outreach activities failed to affect some of the maternal and newborn care practices, and to develop and test practical solutions for addressing them.

We estimated the impact on neonatal mortality of the improvements in maternal and newborn care practices using the Lives Saved Tool (*LiST*) [35]. Through improved coverage of tetanus toxoid vaccination, antenatal care, skilled birth attendance, post-natal care, clean birth practices, and exclusive breastfeeding of neonates over the analysis period, the estimated neonatal mortality rate would decline by approximately 5%, from 38 to 36 deaths per 1,000 live births.

Since late 2011 integrated refresher training for HEWs has been implemented throughout the country, including the essential newborn care practices described here as well as community case management of childhood illnesses [8]. The *LiST* analysis indicates that the scale-up of the essential newborn care package to national level would mean about 7,500 neonatal deaths avoided each year, contributing towards improved child survival. Another recent development of the HEP involves a social mobilization initiative called the ‘health development army’ (Table 1). Women are organized into a group of 30, empowered to learn about the HEP from each other’s experience, with subgroups of six women led by ‘model families’ to form a network. This network increases the density of volunteers and is a tool to increase uptake of services. While one CHP was responsible for providing health education to 25 to 30 households, one health development army member is responsible for the same for five households, across the whole country. Our results on the effect of household visits by CHPs on maternal and newborn care practices suggest that the health development army initiative is likely to be an effective strategy for improving maternal and newborn health practices. Major national efforts to improve maternal mortality currently include mobilizing communities to encourage pregnant mothers to give birth in health facilities; creating effective supportive and referral linkages within the primary health care units; staffing health centers with midwives to ensure continuous availability of basic emergency obstetric care services, and the provision of ambulances to *woredas* to mitigate transportation barriers.

Better maternal and newborn care practices are necessary for improving newborn survival at scale, and they may also pave the way for further interventions. Applying chlorhexidine to the umbilical cord stump to prevent sepsis [36], and community-based newborn sepsis case management [5], are both under consideration by the government.

In conclusion, this study suggests that the integration of community-based essential newborn care package within the HEP, through integrated refresher training of the HEWs, would have a measureable impact on newborn survival. Among the strategic elements of the outreach activities the use of the FHC has been the most effective; however, not all rural households have a FHC, and the HEP should address this gap. Utilizing a network of CHPs to extend the reach of the HEWs was also found as an effective strategy. The ‘health development army’ is thus likely to be a promising strategy to mobilize communities to improve maternal and newborn health. Lastly, a refresher training of the ‘model family’ should be initiated so that they are well aware of the essential newborn care practices.

The HEP outreach activities had little effect on institutional and skilled deliveries for which higher level service providers, supporting technical staff, infrastructure, equipments, supplies, and including a functional referral system, are required. The Government of Ethiopia is taking appropriate measures to strengthen the health systems to ensure universal access to skilled delivery care. Implementation research can support this, to identify the roles of providers within the primary health care unit to maximize the utilization of services that are being made available.
